# Characteristics of Normalization Methods in Quantitative Urinary Metabolomics—Implications for Epidemiological Applications and Interpretations

**DOI:** 10.3390/biom12070903

**Published:** 2022-06-28

**Authors:** Tianqi Li, Tuulia Tynkkynen, Andrei Ihanus, Siyu Zhao, Ville-Petteri Mäkinen, Mika Ala-Korpela

**Affiliations:** 1 Systems Epidemiology, Faculty of Medicine, University of Oulu, 90014 Oulu, Finland; tianqi.li@oulu.fi (T.L.); tuulia.tynkkynen@uef.fi (T.T.); andrei.ihanus@oulu.fi (A.I.); siyu.zhao@oulu.fi (S.Z.); ville-petteri.makinen@sahmri.com (V.-P.M.); 2 Center for Life Course Health Research, Faculty of Medicine, University of Oulu, 90014 Oulu, Finland; 3 Biocenter Oulu, University of Oulu, 90014 Oulu, Finland; 4 NMR Metabolomics Laboratory, School of Pharmacy, University of Eastern Finland, 70210 Kuopio, Finland; 5 Computational and Systems Biology Program, Precision Medicine Theme, South Australian Health and Medical Research Institute, Adelaide, SA 5000, Australia; 6 Australian Centre for Precision Health, Unit of Clinical and Health Sciences, University of South Australia, Adelaide, SA 5000, Australia

**Keywords:** biomarkers, disease risk, epidemiology, kidney function, metabolomics, NMR, normalization, urine

## Abstract

A systematic comparison is presented for the effects of seven different normalization schemes in quantitative urinary metabolomics. Morning spot urine samples were analyzed with nuclear magnetic resonance (NMR) spectroscopy from a population-based group of 994 individuals. Forty-four metabolites were quantified and the metabolite–metabolite associations and the associations of metabolite concentrations with two representative clinical measures, body mass index and mean arterial pressure, were analyzed. Distinct differences were observed when comparing the effects of normalization for the intra-urine metabolite associations with those for the clinical associations. The metabolite–metabolite associations show quite complex patterns of similarities and dissimilarities between the different normalization methods, while the epidemiological association patterns are consistent, leading to the same overall biological interpretations. The results indicate that, in general, the normalization method appears to have only minor influences on standard epidemiological regression analyses with clinical/physiological measures. Multimetabolite normalization schemes showed consistent results with the customary creatinine reference. Nevertheless, interpretations of intra-urine metabolite associations and nuanced understanding of the epidemiological associations call for comparisons with different normalizations and accounting for the physiology, metabolism and kidney function related to the normalization schemes.

## 1. Introduction

Urine is a waste biofluid resulting from the continuous filtration of blood plasma by the kidneys [1,2,3]. Urinary metabolites reflect a plethora of endogenous and exogenous pathways in relation to (patho)physiology, lifestyle, gut microbiome, and short-term food consumption [2,4,5,6,7]. Urine has some advantages in epidemiological studies, for example, it is abundant and non-invasively obtainable with common clinical and laboratory procedures. There is an increased interest in urinary metabolomics; quantitative metabolomics platforms already exist both in nuclear magnetic resonance (NMR) spectroscopy [3,7,8] and in mass spectrometry [9,10], but quantitative applications in epidemiology at appropriate scale are still scarce [3,8,11,12]. The most important issue, however, is that urinary metabolites may provide metabolic information related to kidney function that cannot be obtained by other means [7].

Urine functions as the body’s metabolic sewage, and thus its chemical properties are not under control, e.g., in contradiction to blood plasma, which is tightly physiologically regulated. This leads to the key problem in urinary metabolite analyses; the volume and metabolite concentrations are hugely variable even within the same individual [3]. Therefore, a normalization process is necessary in urinary metabolomics to account for this variation. The use of 24-h urine collections would greatly, and morning spot urine samples partly, reduce the extent of the problem [13], but these types of urinary collections are often not feasible.

The gold standard normalization method is the use of urinary creatinine concentrations as the reference [3,8,9,14]. This relies on the facts that, at a wide range of glomerular filtration rates (GFR), the individual plasma creatinine concentration is almost constant; creatinine is freely filtered and not reabsorbed in the kidneys. GFR reflects the flow of filtered fluid (from blood) through the kidney before water recovery. Hence GFR is not confounded by the incidental variation in urine volume. Referencing to urinary creatinine calibrates the metabolite concentrations to GFR, thus excluding the confounding from urinary volume regulation [1]. Nevertheless, circulating creatinine concentrations are affected by muscle mass and a small amount is secreted by the proximal tubule, resulting in potential case-dependent biases [1,15]. Therefore, various other normalization approaches have been proposed, such as the constant sum (CS) normalization [12] and the probabilistic quotient normalization (PQN) [16]. However, as far as we are aware, no systematic comparison of various normalization methods in quantitative urinary metabolomics is available in an epidemiological setting. Thus, we set up this study with the key goals (1) to understand how various normalization protocols for quantitative urinary metabolomics data compare to the customary normalization to urinary creatinine and (2) to find a rationale for an appropriate normalization strategy for urinary metabolite concentrations to be used in epidemiological analyses.

## 2. Materials and Methods

### 2.1. Study Population and Ethics Approval

The Northern Finland Birth Cohort of 1966 (NFBC66) included 12058 children born alive into the cohort, comprising 96% of all births during 1966 in the region [17]. Data collection in 2012 included clinical examination and urine sampling at the age of 46 years for 4549 individuals. The research protocols were approved by the Ethics Committee of the Faculty of Medicine, University of Oulu and the Ethics Committee of Northern Ostrobothnia Hospital District, Finland. All clinical investigations were conducted according to the principles expressed in the Declaration of Helsinki. Informed written consent was obtained from all participants. More information on the cohort and the 2012 data collection can be found at https://www.oulu.fi/nfbc/ accessed on 30 August 2021.

### 2.2. Urine Sample Collection and Preparation

Urine was collected after an overnight fast using standard clinical protocols. In these morning spot urine samples the biological variability is relatively reduced because of the similar time of accumulation and similar fasting physiological conditions in all the participants [13]. These samples thus allow a good basis to compare the various normalization methods also to the absolute (non-normalized) urinary metabolite concentrations.

Urine samples for the NMR spectroscopy were prepared as previously described [3]. Briefly, urine samples were stored at −80 °C (Thermo Fisher Scientific, Vantaa, Finland) prior to analysis. Before sample preparation, urine samples were thawed overnight in a refrigerator (+4 °C). The thawed samples were gently mixed and centrifuged (3500× *g*, 5 min, +4 °C). A liquid handler, JANUS 8-tip Automated Workstation (PerkinElmer, Turku, Finland) was used to mix 70 µL of phosphate buffer (1.5 M potassium dihydrogen phosphate (Merck, Espoo, Finland), 0.2% sodium azide (Sigma-Aldrich, Espoo, Finland), 5.8 mM 3-(trimethylsilyl)propionic-2,2,3,3-d4 acid sodium salt (TSP) (Eurisotop, Saint-Aubin, France) in deuterium oxide (Eurisotop, Saint-Aubin, France), pH 7.0) and 630 µL of urine in a 96 deep well plate. After centrifugation (3500× *g*, 5 min, +4 °C), 520 µL of each sample was transferred to 5 mm NMR tubes.

### 2.3. NMR Measurements

The detailed measurement protocol has been previously published [3]. Shortly, the urine NMR data were measured with Bruker AVANCE III HD (Bruker BioSpin GmbH, Karlsruhe, Germany) 600 MHz spectrometer equipped with a Bruker Prodigy TCI cryoprobe and an automatic cooled SampleJet sample changer. Standard water suppressed spectra (noesygppr1d) were measured using the following parameters: number of scans 16, spectral width 21.0297 ppm, size of fid 81,920, acquisition time 3.2440 s, relaxation delay 2.5 s, and receiver gain 57. The spectra were measured at 295 K. The spectra were processed and phase-corrected in an automated fashion. The free induction decays were zero-filled to 128 k data points and multiplied with an exponential window function with a 0.3 Hz line broadening.

### 2.4. Metabolite Quantification of the Urine NMR Spectra

The quantification of metabolites from the ^1^H NMR spectra rely on sophisticated lineshape fitting analysis tools developed for high-precision quantitative NMR spectroscopy [18]. In the quantification process, the structures of the multiplets are given in the form of linear equations and prior knowledge for the signal structures or shapes is utilized, which is called constrained total-line-shape (CTLS) fitting [19]. TSP was used as an internal concentration reference and a total of 44 metabolites were quantified as discussed in detail previously [3].

### 2.5. Urinary Metabolomics and Clinical Data

The quantified 44 metabolites are the same as in our previous work [3], but here we have clarified their metabolic classification. Metabolites mainly related to amino acid metabolism were classified as such. Those related to glycolysis and citrate cycle, as well as pentose and glucuronate interconversions, were combined under carbohydrate metabolism. Purine and pyrimidine metabolism were grouped under nucleotide metabolism. Based on recent findings, we also incorporated 2-hydroxyisobutyrate to histone modifications [20] indoxyl sulfate [21] to microbial metabolism and further 2-furoylglycine [22], 3-methylhistidine [23], arabinose [24], sucrose [25] and xylose [24] were classified as dietary metabolites. This led to a new classification, with improved chemical taxonomy and physiological characteristics of the urinary metabolites, with nine metabolic classes: (1) amino acids, (2) related to the metabolism of amino acids, (3) carbohydrate metabolism, (4) nucleotide metabolism, (5) nicotinate and nicotinamide metabolism, (6) microbial metabolism, (7) modification of histones, (8) dietary metabolites, and (9) miscellaneous for those metabolites that did not clearly fall into any of the other categories. It should be noted that these classifications are only suggestive and done mainly from the organizational standpoint since most urinary metabolites are involved in various key metabolic pathways.

In this study, body mass index (BMI) and mean arterial pressure (MAP = (systolic blood pressure + 2 × diastolic blood pressure)/3) were chosen as exemplars of physiological measures to compare the effects of different normalization methods on the associations of the urinary metabolites in typical epidemiological regression analyses.

Quantitative metabolomics data for the 44 urinary metabolites together with the clinical data (sex, BMI, and MAP) were available for 994 individuals.

### 2.6. Overview of the Applied Normalization Methods

In the morning spot urine samples, the absolute urinary metabolite concentrations are also relevant to analyze [13] and are systematically compared here to those of the various normalized concentrations in the correlation and regression analyses.

We applied the following referencing methods: normalization to an internal standard (IS), a constant sum (CS) normalization [26], the probabilistic quotient normalization (PQN) [16], and the DESeq2 method [27]. The fundamental characteristics of these methods are summarized below. Their biological rationales, together with their pros and cons with respect to epidemiological studies, are outlined in Table 1.

**IS-CREA, IS-GLUC, IS-UREA and IS-PSEURID:** The absolute concentration of each urinary metabolite is divided by the concentration of the internal reference metabolite. A widespread convention is to use the creatinine concentration as the internal standard (IS-CREA) [1]. In addition to creatinine, we also tested normalization to glucose (IS-GLUC) [7], urea (IS-UREA) [28], and pseudouridine (IS-PSEURID) [29].

**CS:** The concentration of each metabolite is divided by the sum concentration of all the quantified metabolites. Here all the 44 quantified metabolites were used. 

**PQN:** This method starts with the assumption that biologically relevant concentration changes influence only a limited number of metabolites, while sample dilution (water volume) affects all metabolite concentrations [16]. First, median relative metabolite concentrations are calculated across all samples as the reference values. Second, the quotients between each metabolite and the corresponding reference value are calculated for each sample. Third, the median quotients across all metabolites are calculated for each sample. In the last step, the median quotient is used as the scaling factor in the same manner as the sum is used in CS. 

**DESeq2:** The DESeq2 method is conceptually similar to PQN, but tailored for RNA-seq analyses [27]. First, the reference concentration of each metabolite is calculated as the geometric mean of the absolute metabolite concentrations across all samples (instead of the median in PQN). The subsequent steps in DESeq2 are the same as in PQN.

**Table 1:** The characteristics of employed normalization methods.

Method	Abbr.	Description	Pros for Epidemiology	Cons for Epidemiology	Ref.
**Absolute concentrations**	**ABS**	Using the original quantified concentrations of the urinary metabolites from the ^1^H NMR spectra, i.e., no normalization method applied.	Original data are preserved; the concentration values are straightforward to interpret. Useful if urine volume is noteworthy, e.g., fluid balance. Good for timed collections (e.g., 24 h urine) where exact amount excreted per time unit can be calculated. Morning spot urine accurate enough to detect large effect sizes in epidemiological studies.	Urinary volume, and thus absolute metabolite concentrations, varies greatly day-to-day and person-to-person. Random spot urine samples are thus likely to be too confounded to use without normalization.	[13]
**Normalization to an internal metabolite standard**	**IS-CREA**	The concentration of each metabolite is divided by the concentration of an internal standard. Here, creatinine, glucose, urea, and pseudouridine were used.	Creatinine comes from non-enzymatic breakdown of creatine phosphate in muscles, it is typically produced at a constant rate, and it is stable and inert in plasma. Creatinine is freely filtered by the kidneys and not reabsorbed. The most applied reference, allows straightforward comparisons between studies and meta-analyses.	The stable excretion of creatinine may not hold in the presence of external stimuli or pathophysiological conditions. Renal filtration and excretion of creatinine depend on circulating creatinine concentrations that are depended on muscle mass—thus, the urinary creatinine concentrations can be biased, e.g., for elderly and between men and women. A small amount of creatinine is secreted by the proximal tubule, resulting in a potentially study-dependent bias.	[1,15,30,31,32]
	**IS-GLUC**		Glucose is freely filtered by the kidneys and mostly reabsorbed. The mechanisms of glucose reabsoption are well-understood. Under normoglycemia, the plasma glucose level can be considered stable, and there is always a detectable amount of glucose in normal urine.	At high plasma glucose concentrations (>10 mmol/L), the tubular reabsorption saturates, triggering a pronounced part of filtered glucose to be excreted into the urine. Also, in normoglycemia the urinary glucose is dependent on the circulating glucose, which is widely variable at the population level and also affected by the fasting/non-fasting status. Urinary glucose concentration is dependent on the glomerular filtration rate; this can cause bias in cohorts with large variation in kidney function.	[7]
	**IS-UREA**		Serum urea is applied as a marker of renal function for routine clinical analysis. Urea is freely filtered by the kidneys and about 50% of it is reabsorbed.	Plasma urea concentrations vary widely depending on protein intake, changes in tissue catabolism, and in various pathological conditions. Urea is a waste product, and its excretion is under partial hormonal regulation resulting in variably large amounts to be excreted into the urine.	[1,28]
	**IS-PSEURID**		Pseudouridine is a low-molecular-mass, water-soluble compound with no significant protein binding in serum. Pseudouridine is freely filtered by the kidneys and not reabsorbed. It is not reutilized or metabolized in the body. Pseudouridine concentrations reflect the whole-body turnover of RNA and its excretion appears constant. Pseudouridine concentrations appear independent of muscle mass.	Pseudouridine concentrations appear related to kidney function with potential associated bias. It is the most common RNA modification with links to and potential variation in multiple metabolic diseases.	[29,33,34]
**Constant sum**	**CS**	The concentration of each metabolite is divided by the total sum of all metabolite concentrations.	A generic algorithm that can be applied to any metabolomics platform without requirements for a specific set of metabolites. Water dilution ideally affects all metabolites equally, thus a linear sum over all metabolites should capture volume factor accurately despite (random) variation in any specific metabolite.	Abundance of urinary metabolites resembles the Pareto distribution; a few abundant molecules (e.g., urea) typically dominate the concentration sum. The distribution of a metabolite is usually fat-tailed, thus extreme values may reduce normalization accuracy. Both issues mean that the benefit of averaging across many metabolites may be lost in real data.	[26,35]
**Probabilistic quotient normalization**	**PQN**	A robust version of the CS principle that addresses the Pareto issue of CS (by standardized abundances) and the outlier issue (by the median estimator).	A generic algorithm that can be applied to any metabolomics platform. While water dilution ideally affects all metabolites equally, it is plausible that only a small subset will be affected by the biological phenomenon under study. The median estimator is not much affected by a few biologically-driven or random metabolites, thus PQN can capture the volume factor accurately in most situations.	All normalized concentrations are interdependent (i.e., if some metabolites go up, then others must go down to maintain balance). This means that undesired correlated variation/confounding across many metabolites may cause normalization artefacts. Since abundances are standardized, including low-abundance metabolites near the detection limit may amplify the impact of measurement noise.	[16,26]
**A method for differential gene expression analysis based on the negative binomial distribution**	**DESeq2**	The DESeq2 is a variant of PQN developed for RNA-seq data. Uses geometric mean instead of the median to standardize abundances.	Same benefits as PQN. Geometric mean is better suited for concentrations with limited numerical precision.	Same downsides as in PQN.	[27]

### 2.7. Statistical Analyses

Spearman’s rank correlations adjusted for sex were calculated between all the quantified urinary metabolites. The results are shown in color-coded heat maps. The heat map based on normalization to urinary creatinine was organized via two-dimensional hierarchical clustering. The other heat maps are presented in the same order of metabolites and clusters for easy visual comparison. Creatinine was added to the last row of the heat maps. The reference metabolite correlations were left blank in their corresponding heat maps. The same rank correlation analyses without sex adjustment were repeated for absolute and all normalized concentrations.

To manage multiple testing over the large set of metabolic measures, we first conducted principal component analysis to determine the effective number of independent variables. Thirty-nine principal components were enough to explain >99% of the variation in the concentrations of the quantified 44 urinary metabolites. Therefore, we set the 5% Bonferroni-adjusted type 1 error threshold at *p* < 0.05/39 = 0.0013.

The potential effects and differences due to the various normalization methods on the epidemiological associations of the urinary metabolite concentrations were analyzed via linear regression models. BMI and MAP were used as exemplars of clinical measures and the models were run with and without adjusting for sex. Before regression analysis, extreme metabolite levels were truncated to third quartile + 8 × interquartile range and then log-transformed. Association magnitudes are reported in standard deviation (SD) units throughout to ease the comparison across multiple measures. All statistical analyses were performed using the R software (version 4.1.2).

## 3. Results

**Figure 1:**
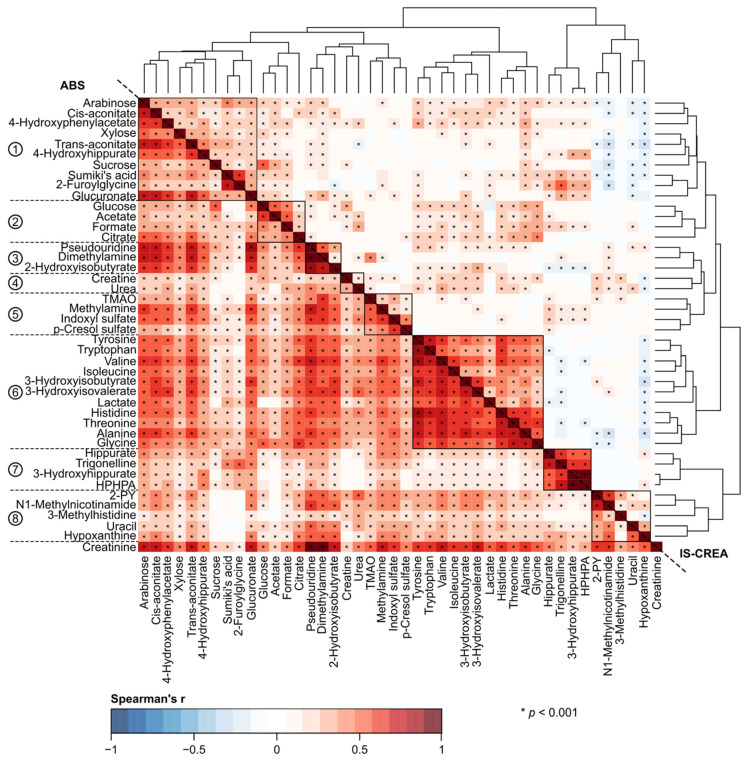
The urinary metabolite–metabolite associations as indicated by Spearman’s rank correlations adjusted for sex. The down-left triangle shows results for the absolute urinary metabolite concentrations (i.e., no normalization applied) and the top-right triangle for the creatinine normalization (IS-CREA). Two-dimensional hierarchical clustering was applied to organize the IS-CREA heat map to make detailed comparisons of the metabolite–metabolite associations feasible between the different normalization schemes. All heat maps are presented in the same order of metabolites with creatinine added to the last row. The reference metabolite correlations are left blank in their corresponding heat maps. As a by-product of the hierarchical clustering, eight urinary metabolite clusters were identified as numbered on the left and detailed in the Results section. Abbreviations: TMAO, trimethylamine N-oxide; HPHPA, 3-(3-hydroxyphenyl)-3-hydroxypropanoate; 2-PY, N1-Methyl-2-pyridone-5-carboxamide.

**Figure 2:**
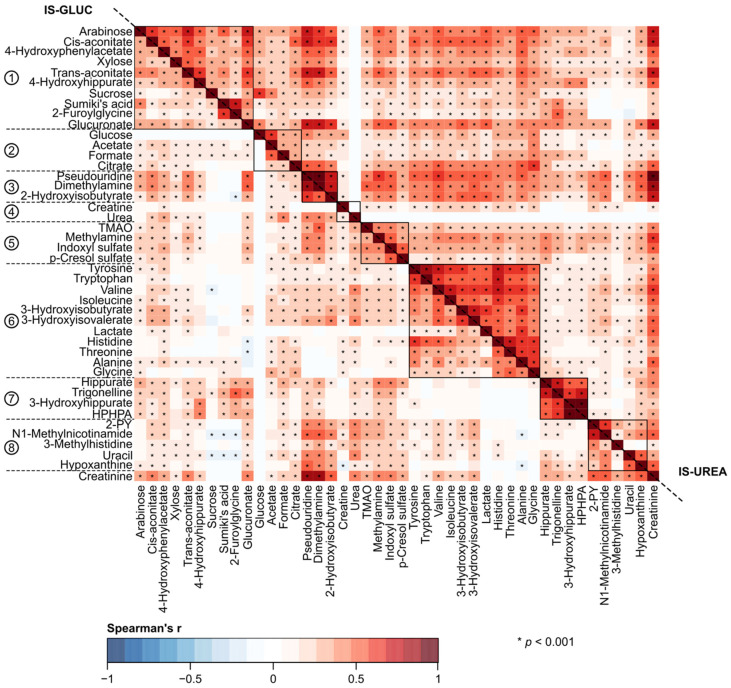
The urinary metabolite–metabolite associations as indicated by Spearman’s rank correlations adjusted for sex. The down-left triangle shows results for the glucose normalization (IS-GLUC) and the top-right triangle for the urea normalization (IS-UREA). The order of metabolites (with creatinine added to the last row) is the same as in Figure 1 and based on the two-dimensional hierarchical clustering of the IS-CREA heat map. The reference metabolite correlations are left blank in their corresponding heat maps. Abbreviations: TMAO, trimethylamine N-oxide; HPHPA, 3-(3-hydroxyphenyl)-3-hydroxypropanoate; 2-PY, N1-Methyl-2-pyridone-5-carboxamide.

**Figure 3:**
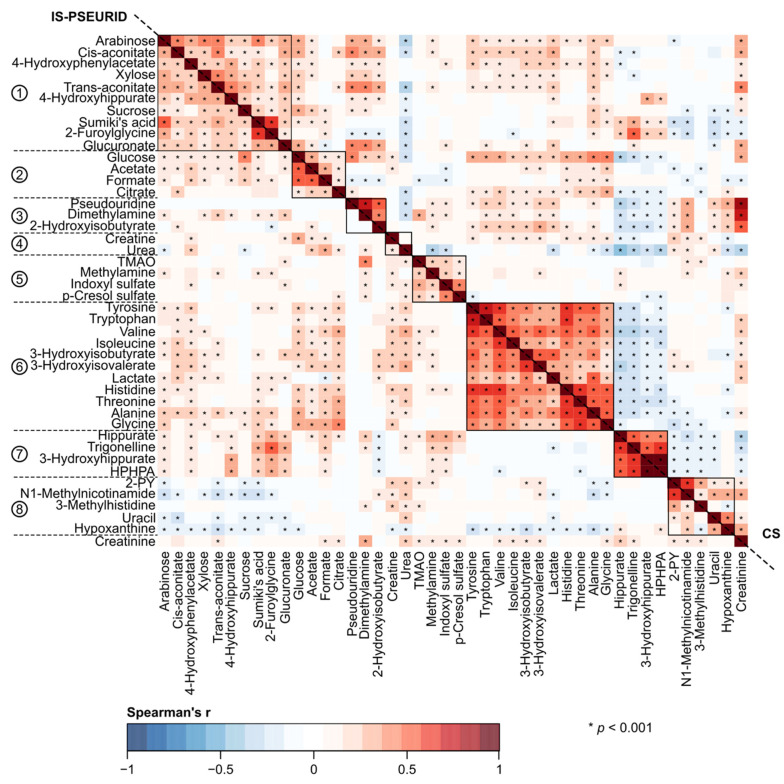
The urinary metabolite–metabolite associations as indicated by Spearman’s rank correlations adjusted for sex. The down-left triangle shows results for the pseudouridine normalization (IS-PSEURID) and the top-right triangle for the constant sum normalization (CS). The order of metabolites (with creatinine added to the last row) is the same as in Figure 1 and based on the two-dimensional hierarchical clustering of the IS-CREA heat map. The reference metabolite correlations are left blank in their corresponding heat maps. Abbreviations: TMAO, trimethylamine N-oxide; HPHPA, 3-(3-hydroxyphenyl)-3-hydroxypropanoate; 2-PY, N1-Methyl-2-pyridone-5-carboxamide.

**Figure 4:**
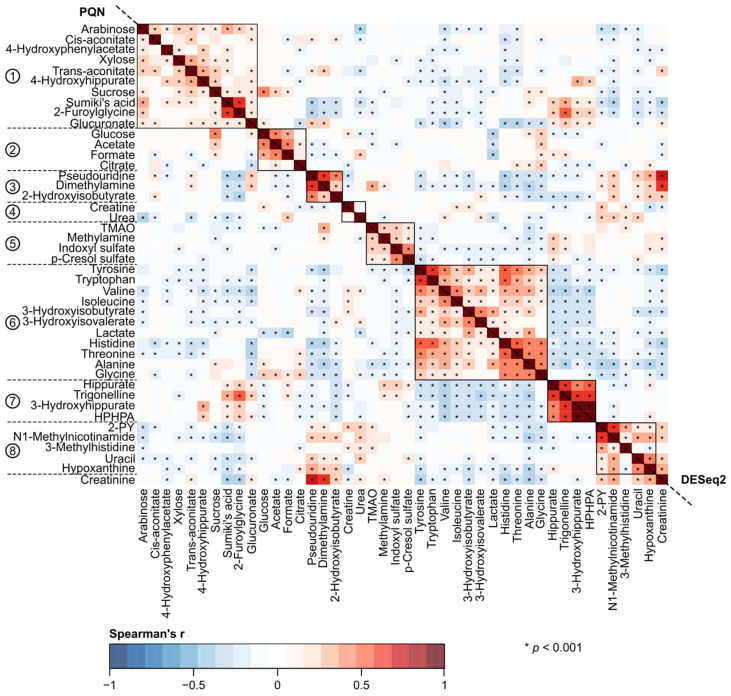
The urinary metabolite–metabolite associations as indicated by Spearman’s rank correlations adjusted for sex. The down-left triangle shows results for the probabilistic quotient normalization (PQN) and the top-right triangle for the DESeq2 normalization. The order of metabolites (with creatinine added to the last row) is the same as in Figure 1 and based on the two-dimensional hierarchical clustering of the IS-CREA heat map. Abbreviations: TMAO, trimethylamine N-oxide; HPHPA, 3-(3-hydroxyphenyl)-3-hydroxypropanoate; 2-PY, N1-Methyl-2-pyridone-5-carboxamide.

**Figure 5:**
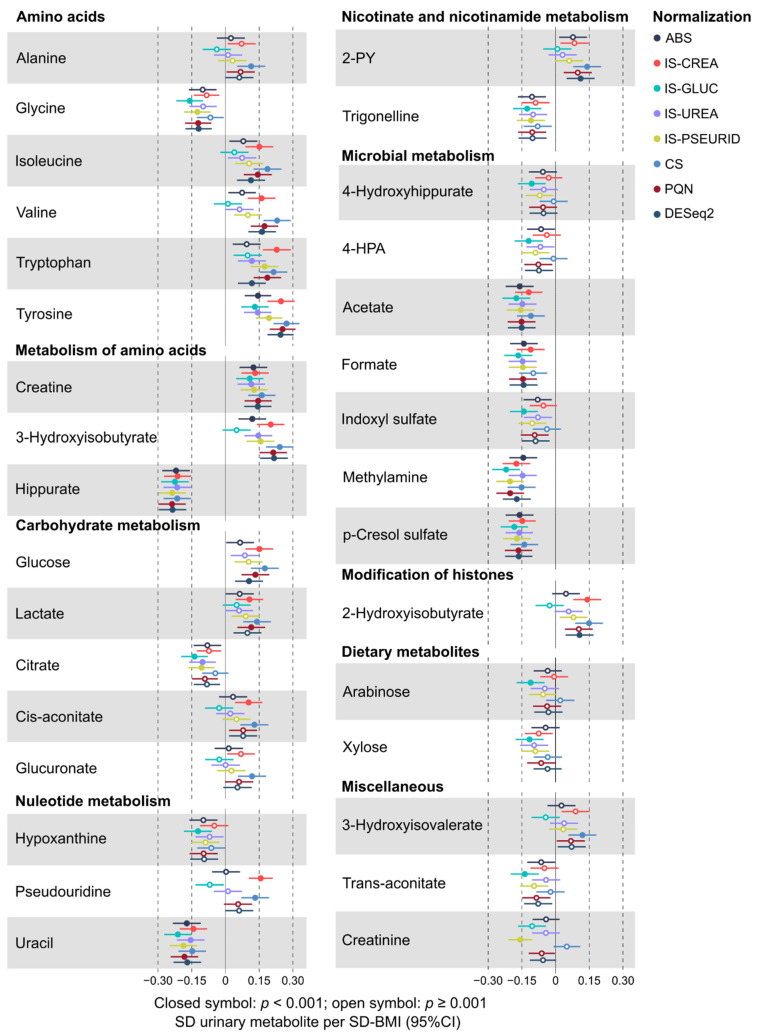
The robust associations (*p* < 0.001) of the urinary metabolite concentrations with BMI for the various normalization schemes. See Table 1 for the explanation and basis of the normalization methods. Abbreviations: 2-PY, N1-Methyl-2-pyridone-5-carboxamide; 4-HPA, 4-Hydroxyphenylacetate.

**Figure 6:**
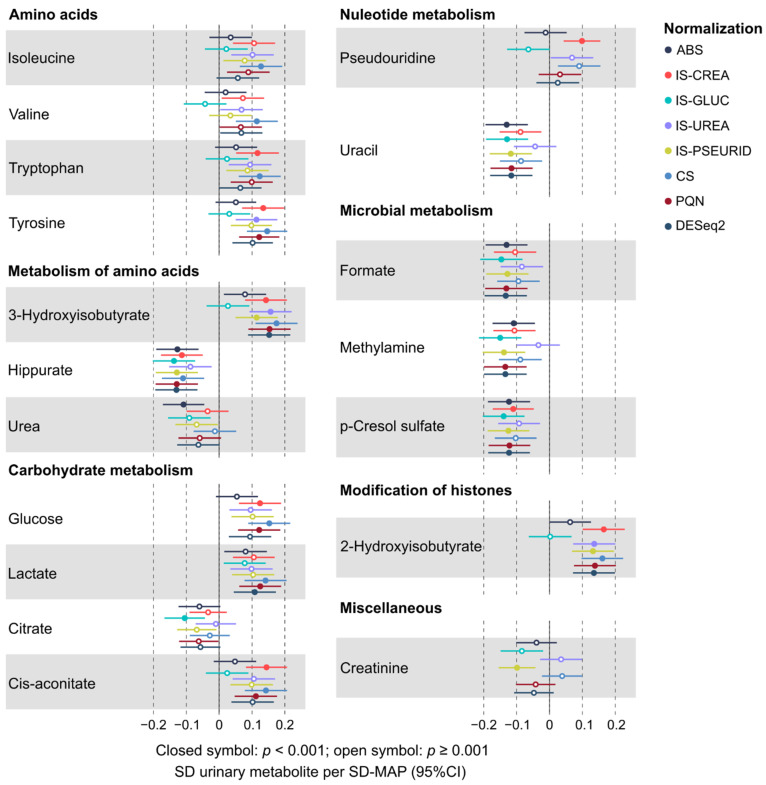
The robust associations (*p* < 0.001) of the urinary metabolite concentrations with MAP for the various normalization schemes. See Table 1 for the explanation and basis of the normalization methods.

A key objective for this work was to understand the effects of various normalization approaches on quantitative urinary metabolomics data. We applied six different normalization methods in comparison to the absolute metabolite concentrations and the customary normalization of the absolute concentrations via urinary creatinine concentration. We investigated and compared the effects of all these approaches in epidemiological analyses. The underlying characteristics of these employed normalization approaches are summarized in Table 1. Figure 1, Figure 2, Figure 3 and Figure 4 illustrate the metabolite–metabolite associations in all these normalization scenarios. Figure 5 and Figure 6 depict how these different normalization scenarios affect the results of epidemiological regression analyses between two types of different physiological measures, namely BMI and MAP, and the urinary metabolites. While these extensive analyses show close similarities between some normalization methods, there are also substantial differences. The results are described in a fair amount of detail below for the urinary metabolite–metabolite associations and the related metabolic cluster analysis (3.1.) and for the epidemiological regression modeling (3.2.). These are then followed by discussion on how these intricate findings should be taken into consideration in the future applications of quantitative urinary metabolomics in epidemiology.

### 3.1. Intra-Urinary Metabolite Associations and Metabolic Cluster Analysis

Figure 1 shows the urinary metabolite–metabolite associations normalized via IS-CREA (top-right), the most common choice to normalize urinary metabolite concentrations, and without any normalization, i.e., using directly the quantified absolute metabolite concentrations (down-left). We applied two-dimensional hierarchical clustering on the associations based on IS-CREA to get a clearer overall view on the metabolite associations. This clustering, i.e., the order of metabolites, was then used in all the visualizations for the other normalization methods to facilitate straightforward visual comparisons.

The general association characteristics are summarised via eight metabolic clusters depicted and numbered on the left in Figure 1. The most pronounced Cluster 6 reflects strong associations between all the quantified amino acids, 3-hydroxyisobutyrate, 3-hydroxyisovalerate, and lactate with abundant positive links to Clusters 1–5 and some sparse negative ones to Clusters 7 and 8. The second biggest Cluster 1 connects together multiple diet-related metabolites (arabinose, xylose, sucrose, and 2-furoylglycine), carbohydrate metabolism (cis-aconitate and glucuronate), microbial metabolism (4-hydroxyphenylacetate and 4-hydroxyhippurate), trans-aconitate and Sumiki’s acid with positive links to all other clusters but Cluster 8 with which it has abundant negative associations. Microbial metabolites 3-hydroxyhippurate and 3-(3-hydroxyphenyl)-3-hydroxypropanoate (HPHPA) form Cluster 7 together with hippurate and trigonelline. Cluster 2 combines carbohydrate metabolism (glucose and citrate) and microbial metabolism (acetate and formate). Cluster 4 combines creatine and urea which are related to amino acids metabolism. Cluster 8 involves 2-PY and N1-methylnicotinamide (nicotinate and nicotinamide metabolism), uracil and hypoxanthine (nucleotide metabolism), and 3-methylhistidine (a diet-related metabolite). The other Clusters 3 and 5 are small and consist of quite heterogeneous mixture of metabolites, though all the metabolites in Cluster 5 can be identified as microbial metabolites.

The broad comparison of all the eight different metabolite association heat maps in Figure 1, Figure 2, Figure 3 and Figure 4 reveals both marked similarities and dissimilarities. The heat maps based on IS-CREA (Figure 1 top-right) and IS-PSEURID (Figure 3 down-left) normalization have a globally similar association structure (please note to mirror through the diagonal when comparing). The same holds for the absolute concentrations (Figure 1 down-left) and IS-UREA (Figure 2 top-right) as well as for the normalizations via PQN and DESeq2 (Figure 4 down-left and top-right, respectively). IS-GLUC (Figure 2 down-left) and the constant sum normalization (Figure 3 top-right) do not very well match with any other overall association pattern.

For the absolute concentrations and for the normalizations via IS-UREA, IS-GLUC, IS-PSEURID, and the constant sum, the associations within the metabolite clusters are mostly strongly positive, i.e., similar to those of IS-CREA normalization used to form the clusters. The overall association patterns for PQN and DESeq2 normalizations are somewhat weaker than for the other normalization methods with respect to many of the metabolite associations within the clusters. The constant sum normalization matches quite closely to PQN and DESeq2 with respect to the overall associations for Clusters 7 and 8, but in contrast, the associations between Cluster 6 and Clusters 1–4 are overall positive. The positive associations between Clusters 1 and 2 as well as 1 and 3 for the constant sum normalization are also contrasting to those for the PQN and DESeq2 normalizations. Regarding these association patterns, the constant sum normalization resembles rather closely that of IS-CREA. The other associations in the PQN and DESeq2 normalized data do not display much resemblance to those of the other normalization methods.

Figure 1, Figure 2, Figure 3 and Figure 4 also depict the associations of creatinine with the other urinary metabolites for all the normalization methods (except IS-CREA). They are very similar for the PQN and DESeq2 normalized data, with more negative than positive associations, and vary somewhat between the other normalization methods for which almost all creatinine associations are positive.

Corresponding heat maps for Figure 1, Figure 2, Figure 3 and Figure 4, but without adjusting for sex, are shown in Supplementary Figures S1–S4. The overall correlation patterns for the absolute urinary metabolite concentration data as well as for all the various normalization approaches are closely similar to those in the sex adjusted analyses.

### 3.2. Epidemiological Exemplars: BMI and MAP

Based on the multiple testing corrected *p*-value threshold of 0.0013, there were 32 and 18 robust associations for sex-adjusted BMI (Figure 5) and MAP (Figure 6) with the urinary metabolites, respectively. The associations with all the quantified urinary metabolites (*n* = 44) are shown for BMI in Supplementary Figure S5 (not adjusted for sex) and Supplementary Figure S6 (adjusted for sex) and for MAP in Supplementary Figure S7 (not adjusted for sex) and Supplementary Figure S8 (adjusted for sex). Urinary metabolite concentrations via all the above-mentioned seven normalization methods as well their absolute concentrations were exploited in the linear regression analyses.

Overall, the associations between the urinary metabolites and BMI are stronger than those between MAP. Regarding both outcomes, robust associations with amino acid and carbohydrate pathways as well as with microbial metabolites are commonplace. While some urinary metabolites—as citrate in the case of BMI and glycine, histidine, tyrosine, lactate, citrate, 4-hydroxyhippurate, and creatinine for MAP—appear more related to sex than others, i.e., the sex adjustment markedly affects the strength of the association, the overall association pattern with respect to all the normalization methods is coherent for all metabolites and both outcomes with or without adjusting for sex.

A prominent feature in Figure 5 and Figure 6 (as well as in the related Supplementary Figures S5–S8) is that, in general, for these epidemiological associations, all the normalized data and often also the absolute urinary metabolite concentrations give coherent results. However, a more meticulous view suggests that the results for the concentrations normalized via IS-GLUC are rather often trending slightly away from those with the other methods. This can be seen, for example, for various amino acid related metabolites and 2-hydroxyisobutyrate for both BMI and MAP. Apart from these deviations for IS-GLUC, and maybe some incidental ones for IS-PSEURID, IS-UREA, and the constant sum normalization methods, the results from these epidemiological regression analyses are strikingly consistent. Particularly, the results for PQN and DESeq2 are almost identical and usually very similar to those for IS-CREA and the constant sum normalization. The similarity of the epidemiological associations with BMI for the urinary metabolite concentrations via IS-CREA and PQN normalization is illustrated in Supplementary Figure S9.

The overall correspondence of the individual metabolite concentrations, not necessarily in an absolute manner but via correlation, is the fundament for the epidemiological correspondence of the normalization methods. Thus, we also calculated mean R^2^-values for the correlations between all the 44 individual metabolite concentrations between all the different normalization methods. These 28 different comparisons are given in the Supplement in Table S1 to provide quantitative support for the above interpretations on the epidemiological association patterns.

## 4. Discussion

We present here the first comprehensive and systematic comparison of the effects of various normalization methods in quantitative urinary metabolomics in an epidemiological setting. In a selection of 994 participants from a population-based epidemiological cohort (NFBC66), we quantified 44 metabolites from the NMR spectra of their morning spot urine samples. We analysed the urinary metabolite–metabolite associations as well as the associations of urinary metabolite concentrations with two physiological measures, BMI and MAP, in seven different normalization schemes. We studied four internal metabolite concentration standards; the most applied normalization to urinary creatinine was accompanied with referencing to urinary glucose, urea, and pseudouridine. In addition, a constant sum normalization, in which a sum of all quantified metabolite concentrations is used as the reference, was tested together with two of its more sophisticated versions, the PQN and the DESeq2. As morning spot urine was studied, we also included a full comparison to the original absolute concentrations of the urinary metabolites in the samples. The biological rationale for each of these normalization options, together with their pros and cons with respect to epidemiological studies, are outlined in Table 1. We would like to emphasize that this work, and the entire development of the urine NMR metabolomics platform, is focusing on large-scale epidemiology and genetics, aiming for an experimental protocol that is high-throughput and cost-effective for tens of thousands of samples in multiple laboratories and in line with epidemiological and clinical sample collection routines [3]. Thus, by definition, some normalization methods, e.g., urine osmolality or dry mass, are not feasible in this case [36,37].

We applied two-dimensional hierarchical clustering to organize the color-coded correlation heat map of urinary metabolite–metabolite concentrations normalized to urinary creatinine (Figure 1). This was done to make detailed comparisons of the metabolite–metabolite associations feasible between the different normalization schemes. We identified eight urinary metabolite clusters and their interrelationships. Though these clusters (and the new improved primary classification of the urinary metabolites presented in the Methods section) have not been directly identified before, we have discussed the intra-fluid metabolic associations in urine already previously [3] and will not focus anymore on this issue here (more than done in the Results section). Along the same lines, many of the detected urinary metabolite associations with blood pressure and particularly with BMI are rather well-known [3,4,5] and are not the focus here but for how they are affected by the different normalization methods.

A rather striking difference is seen when comparing the effects of normalization for the intra-urine metabolite associations (Figure 1, Figure 2, Figure 3 and Figure 4) with those for the epidemiological associations via the regression models (Figure 5 and Figure 6). While the metabolite–metabolite associations show quite complex patterns of similarities and dissimilarities for the different normalization methods (as detailed in the Results section), the epidemiological association patterns for both BMI and MAP with all the metabolites are consistent. There are marked differences in both in the direction and strength of the associations between the normalized urinary metabolite concentrations. However, almost all the epidemiological associations, with respect to all the normalization methods, would have the same overall biological interpretation, i.e., the same direction of association though there are some variations in the strength of the associations. Only very few point estimates are contradicting regarding the direction of the associations between the urinary metabolite concentrations and the clinical measures.

As detailed in Table 1, both urinary glucose [7] and urea [1,28] have physiology-related limitations for being optimal individual metabolite references. Comparison of the metabolite–metabolite associations for these normalizations (Figure 2) to those with original absolute metabolite concentrations (Figure 1) indicates close similarity and mainly positive associations between the metabolites. This suggests that IS-GLUC and IS-UREA normalized metabolite concentrations still contain a rather large component related to the overall volume and dilution of the urine samples.

While urinary creatinine [1,15,30,31,32] and pseudouridine [29,33,34] have quite a different biological background and limitations as a reference molecule (Table 1), their metabolite–metabolite correlation heat maps are almost identical (Figure 1 top-right and Figure 3 down-left, respectively). IS-PSEURID might thus be a good normalization scheme to use to check consistency of the IS-CREA results under different biological assumptions.

Multimetabolite normalization schemes have previously been developed according to the viewpoint that summing over multiple metabolites should balance the variation in the individual metabolites and increase the robustness of the correction for the variable dilution in the urine samples [26,35]. The three such schemes applied here—the constant sum normalization, the PQN, and the DESeq2—produced a complicated pattern of similarities and dissimilarities for the metabolite–metabolite associations but were very coherent in the epidemiological regression analyses for both clinical measures. Their point estimates were often very close to those of the IS-CREA normalized metabolite concentrations. The corollary for epidemiological studies would be, specifically when many individual quantitative metabolites are available, to use these multimetabolite normalization schemes to check consistency of the IS-CREA results and to assess if they could strengthen some associations due to potentially better accounting for the biologically relevant concentration changes of the urinary metabolites (Table 1).

Urine is arguably the main biological material that is collected to study kidney diseases, and these diseases typically alter the secretion and excretion of creatinine, thus undermining the viability of the IS-CREA method. On the other hand, the quantile-based DESeq2 method has become de facto choice for RNA profiling, where internal standards like creatinine are unavailable. For this reason, we recommend including, and maybe relying more on, the PQN method in situations where it is plausible that the creatinine measurement itself is inaccurate or compromised by pathophysiological factors.

We would like to emphasize that this is a cross-sectional epidemiological data set with a limited number of people. However, for the purpose of the study, as demonstrated by the robustness of the multiple testing-corrected results for all the analyses performed, the data are ample. As always in epidemiological studies, potential cohort-related biases for the results cannot be dissected and care must be taken in extrapolating the results. Most of the urinary metabolite associations with the clinical measures replicated previous findings in other cohorts and data sets. It is thus likely that the key findings regarding the effects of the various normalization schemes would be generally valid. Alongside independent data coming available, replication of the findings would of course be preferable. In addition, it would be good to keep in mind that the participants studied in this work were a selection of a birth cohort study at an average age of 46 years. They thus represent an apparently rather healthy group of individuals with only rather minor changes in kidney and other organ and metabolic functions. Thus, these results should not be extrapolated to individuals or patients with marked organ or metabolic dysfunction.

## 5. Conclusions

The results of this comprehensive and systematic comparison of the effects of seven different normalization schemes (together with the absolute non-normalized metabolite concentrations) in quantitative urinary metabolomics can be encapsulated by saying that the effects and differences of data normalization are pronounced for the urinary metabolite–metabolite associations but markedly consistent for epidemiological regression analyses with clinical measures. Thus, particularly for epidemiological studies on the role of urinary metabolites, the custom of using urinary creatinine as the concentration reference appears sensible. Normalization to urinary pseudouridine might be useful to check consistency of the creatinine-referenced results under different biological assumptions. If many individual quantitative metabolites are available, multimetabolite normalization schemes might also be worth applying to check consistency of the creatinine-referenced results and, in addition, to assess if they could strengthen some associations due to potentially better accounting for the biologically relevant concentration changes of the urinary metabolites. Comparison of different normalization schemes while also taking physiology, metabolism, and kidney function into consideration would likely be beneficial for detailed interpretations of intra-urine metabolite associations and when aiming for nuanced elucidation of the urinary metabolite associations in epidemiological studies.

## Data Availability

The NFBC data used in the current study are available through an application process for researchers who meet the criteria to access confidential data: https://www.oulu.fi/nfbc/, accessed on 30 August 2021.

## Supplementary Materials

The following are available online at https://www.mdpi.com/article/10.3390/biom12070903/s1, Table S1: The mean R^2^-values for the correlations between all the 44 individual urinary metabolite concentrations between all the different normalization methods. Figure S1: The urinary metabolite–metabolite associations as indicated by Spearman’s rank correlations without adjustments for the absolute urinary metabolite concentrations and the IS-CREA normalization. Figure S2: The urinary metabolite–metabolite associations as indicated by Spearman’s rank correlations without adjustments for the IS-GLUC and the IS-UREA normalizations. Figure S3: The urinary metabolite–metabolite associations as indicated by Spearman’s rank correlations without adjustments for the IS-PSEURID and the CS normalizations. Figure S4: The urinary metabolite–metabolite associations as indicated by Spearman’s rank correlations without adjustments for the PQN and the DESeq2 normalizations. Figure S5: The associations of the urinary metabolite concentrations with BMI (without adjustment) for the various normalization schemes. Figure S6: The associations of the urinary metabolite concentrations with BMI (adjusted for sex) for the various normalization schemes. Figure S7: The associations of the urinary metabolite concentrations with MAP (without adjustment) for the various normalization schemes. Figure S8: The associations of the urinary metabolite concentrations with MAP (adjusted for sex) for the various normalization schemes. Figure S9: Comparison of the epidemiological associations of the urinary metabolite concentrations via IS-CREA and PQN normalization with BMI.
