# Supplementary material for: Characteristics of Normalization Methods in Quantitative Urinary Metabolomics—Implications for Epidemiological Applications and Interpretations
Source: Biomolecules. 2022 Jun 28;12(7):903. doi: 10.3390/biom12070903 (PMC9313036; doi:10.3390/biom12070903)
Supplement: Supplementary file 1 [file biomolecules-12-00903-s001.zip › biomolecules-1758882-supplementary.pdf]

Supplementary Materials

Table S1. The mean R^2-values for the correlations between all the 44 individual urinary metabolite concentrations between all the different normalization methods.

Figure S1. The urinary metabolite-metabolite associations as indicated by Spearman's rank correlations without adjustments. The down-left triangle shows results for the absolute urinary metabolite concentrations (i.e., no normalization applied) (ABS) and the top-right triangle for the creatinine normalization (IS-CREA).

Figure S2. The urinary metabolite-metabolite associations as indicated by Spearman's rank correlations without adjustments. The down-left triangle shows results for the glucose normalization (IS-GLUC) and the top-right triangle for the urea normalization (IS-UREA).

Figure S3. The urinary metabolite-metabolite associations as indicated by Spearman's rank correlations without adjustments. The down-left triangle shows results for the pseudouridine normalization (IS-PSEURID) and the top-right triangle for the constant sum normalization (CS).

Figure S4. The urinary metabolite-metabolite associations as indicated by Spearman's rank correlations without adjustments. The down-left triangle shows results for the probabilistic quotient normalization (PQN) and the top-right triangle for the DESeq2 normalization.

Figure S5. The associations of the urinary metabolite concentrations with BMI (without adjustments) for the various normalization schemes.

Figure S6. The associations of the urinary metabolite concentrations with BMI (adjusted for sex) for the various normalization schemes.

Figure S7. The associations of the urinary metabolite concentrations with MAP (without adjustments) for the various normalization schemes.

Figure S8. The associations of the urinary metabolite concentrations with MAP (adjusted for sex) for the various normalization schemes.

Figure S9. Comparison of the epidemiological associations of the urinary metabolite concentrations via IS-CREA and PQN normalization with BMI.

Table S1. The mean R^2-values for the correlations between all the 44 individual urinary metabolite concentrations between all the different normalization methods.

	IS-CREA	IS-GLUC	IS-UREA	IS-PSEURID	CS	PQN	DESeq2
**ABS**	0.53 ± 0.25	0.35 ± 0.26	0.80 ± 0.10	0.55 ± 0.23	0.68 ± 0.18	0.58 ± 0.23	0.59 ± 0.23
**IS-CREA**		0.57 ± 0.24	0.55 ± 0.23	0.93 ± 0.05	0.79 ± 0.17	0.85 ± 0.15	0.84 ± 0.15

**IS-GLUC**			0.37 ± 0.26	0.59 ± 0.23	0.56 ± 0.24	0.65 ± 0.22	0.64 ± 0.22
**IS-UREA**				0.53 ± 0.23	0.70 ± 0.16	0.57 ± 0.23	0.58 ± 0.22
**IS-PSEURID**					0.77 ± 0.17	0.86 ± 0.13	0.86 ± 0.13
**CS**						0.87 ± 0.12	0.87 ± 0.12
**PQN**							0.99 ± 0.01

The values represent the mean R^2 of all the 44 quantified urinary metabolites ± SD in each corresponding comparison.

Please see Table 1 for the description and abbreviations of the normalization methods.

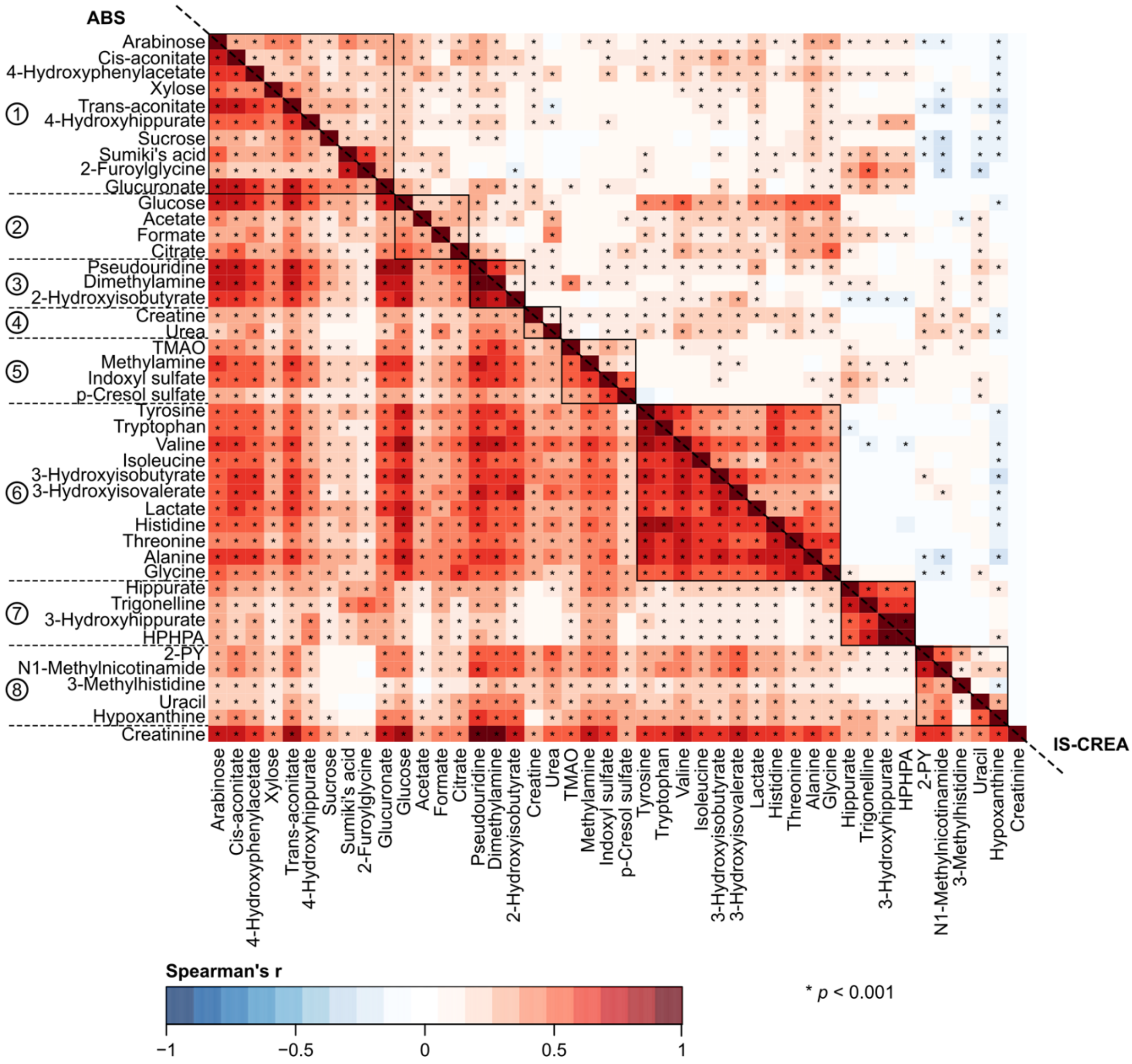

Figure S1. The urinary metabolite-metabolite associations as indicated by Spearman's rank correlations without adjustments. The down-left triangle shows results for the absolute urinary metabolite concentrations (i.e., no normalization applied) (ABS) and the top-right triangle for the creatinine normalization (IS-CREA). All heat maps are presented in the same order of metabolites with creatinine added to the last row. The reference metabolite correlations are left blank in their corresponding heat maps. The order of metabolites (with creatinine added to the last row) is the same as in Figure 1 which based on the two-dimensional hierarchical clustering of the IS-CREA (adjusted for sex) heat map. Abbreviations: TMAO, trimethylamine N-oxide; HPHPA, 3-(3-hydroxyphenyl)-3-hydroxypropanoate; 2-PY, N1-Methyl-2-pyridone-5-carboxamide.

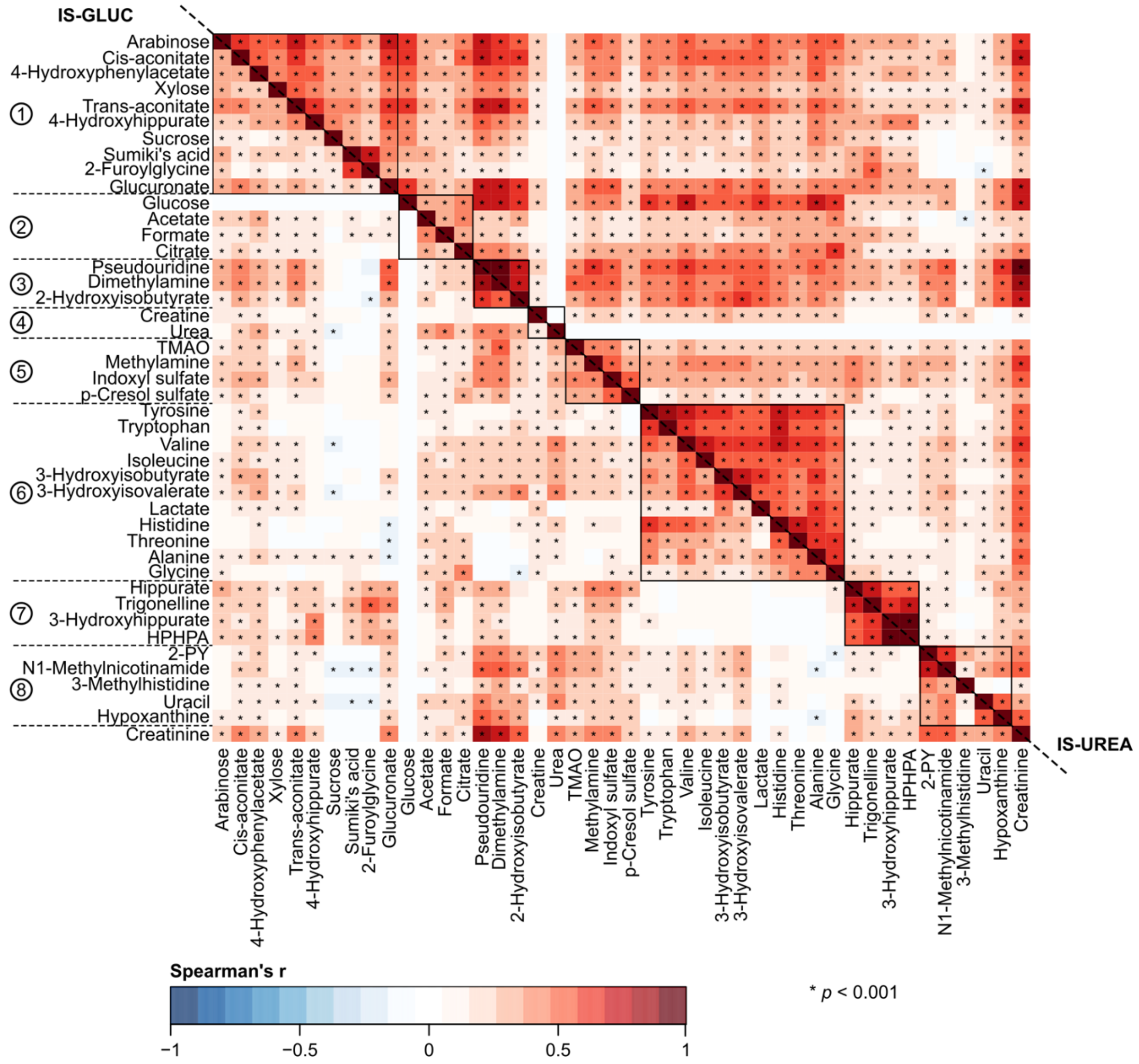

Figure S2. The urinary metabolite-metabolite associations as indicated by Spearman's rank correlations without adjustments. The down-left triangle shows results for the glucose normalization (IS-GLUC) and the top-right triangle for the urea normalization (IS-UREA). The order of metabolites (with creatinine added to the last row) is the same as in Figure 1 which based on the two-dimensional hierarchical clustering of the IS-CREA (adjusted for sex) heat map. The reference metabolite correlations are left blank in their corresponding heat maps. Abbreviations: TMAO, trimethylamine N-oxide; HPHPA, 3-(3-hydroxyphenyl)-3-hydroxypropanoate; 2-PY, N1-Methyl-2-pyridone-5-carboxamide.

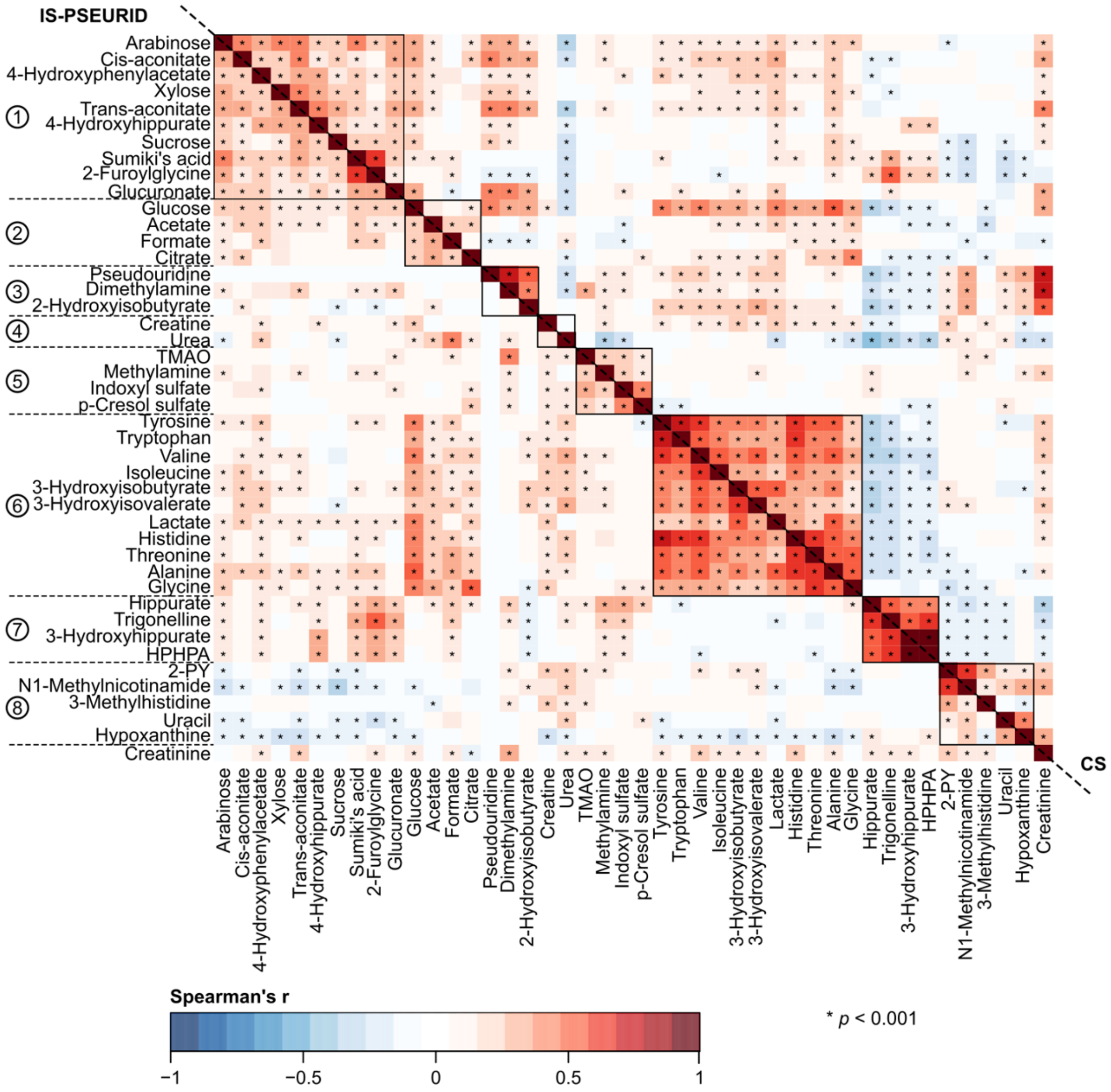

Figure S3. The urinary metabolite-metabolite associations as indicated by Spearman's rank correlations without adjustments. The down-left triangle shows results for the pseudouridine normalization (IS-PSEURID) and the top-right triangle for the constant sum normalization (CS). The order of metabolites (with creatinine added to the last row) is the same as in Figure 1 which based on the two-dimensional hierarchical clustering of the IS-CREA (adjusted for sex) heat map. The reference metabolite correlations are left blank in their corresponding heat maps. Abbreviations: TMAO, trimethylamine N-oxide; HPHPA, 3-(3-hydroxyphenyl)-3-hydroxypropanoate; 2-PY, N1-Methyl-2-pyridone-5-carboxamide.

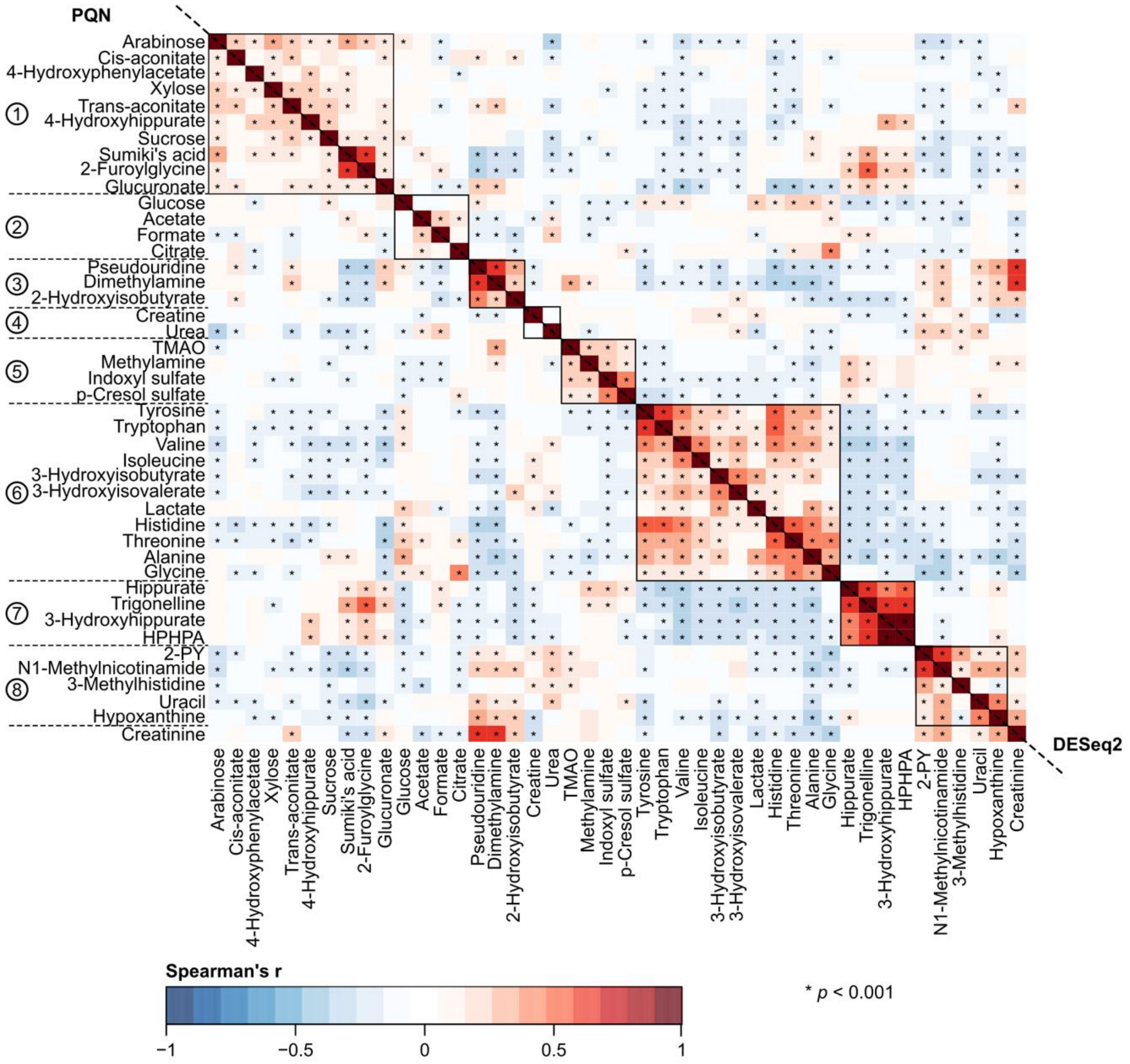

Figure S4. The urinary metabolite-metabolite associations as indicated by Spearman's rank correlations without adjustments. The down-left triangle shows results for the probabilistic quotient normalization (PQN) and the top-right triangle for the DESeq2 normalization. The order of metabolites (with creatinine added to the last row) is the same as in Figure 1 which based on the two-dimensional hierarchical clustering of the IS-CREA (adjusted for sex) heat map. Abbreviations: TMAO, trimethylamine N-oxide; HPHPA, 3-(3-hydroxyphenyl)-3-hydroxypropanoate; 2-PY, N1-Methyl-2-pyridone-5-carboxamide.

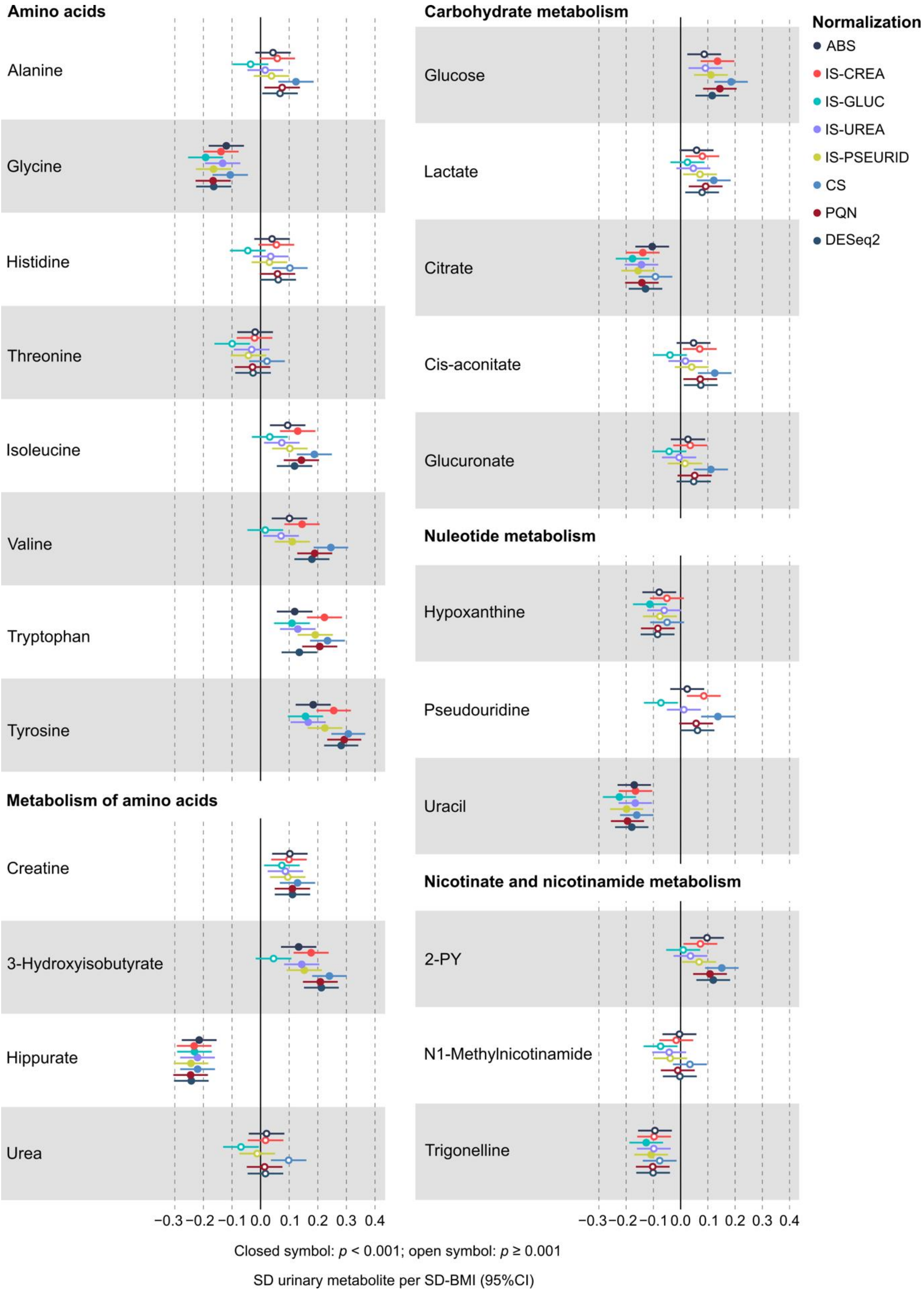

Continued on the next page

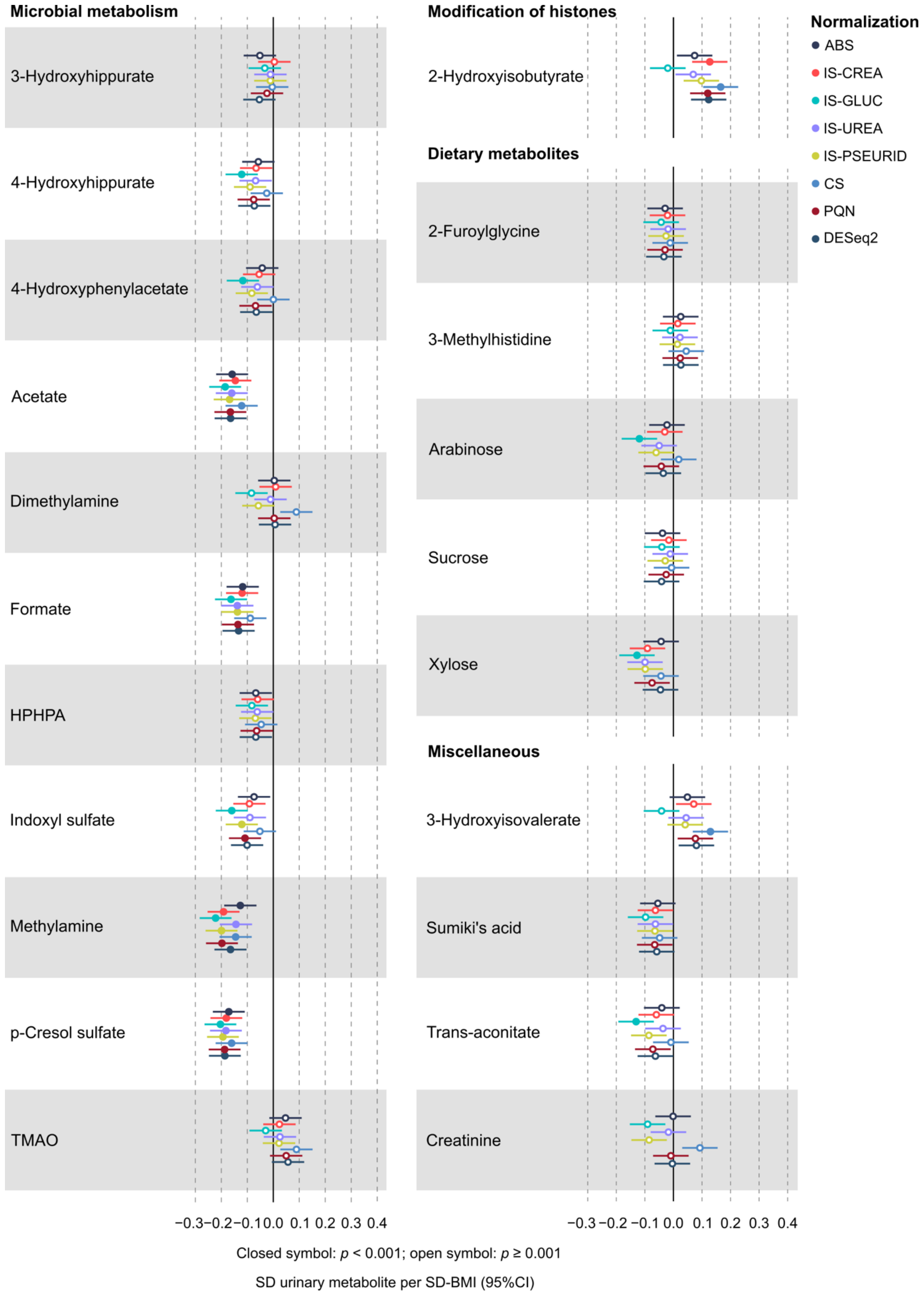

Figure S5. The associations of the urinary metabolite concentrations with BMI (without adjustments) for the various normalization schemes. Abbreviations: TMAO, trimethylamine N-oxide; HPHPA, 3-(3-hydroxyphenyl)-3-hydroxypropanoate; 2-PY, N1-Methyl-2-pyridone-5-carboxamide.

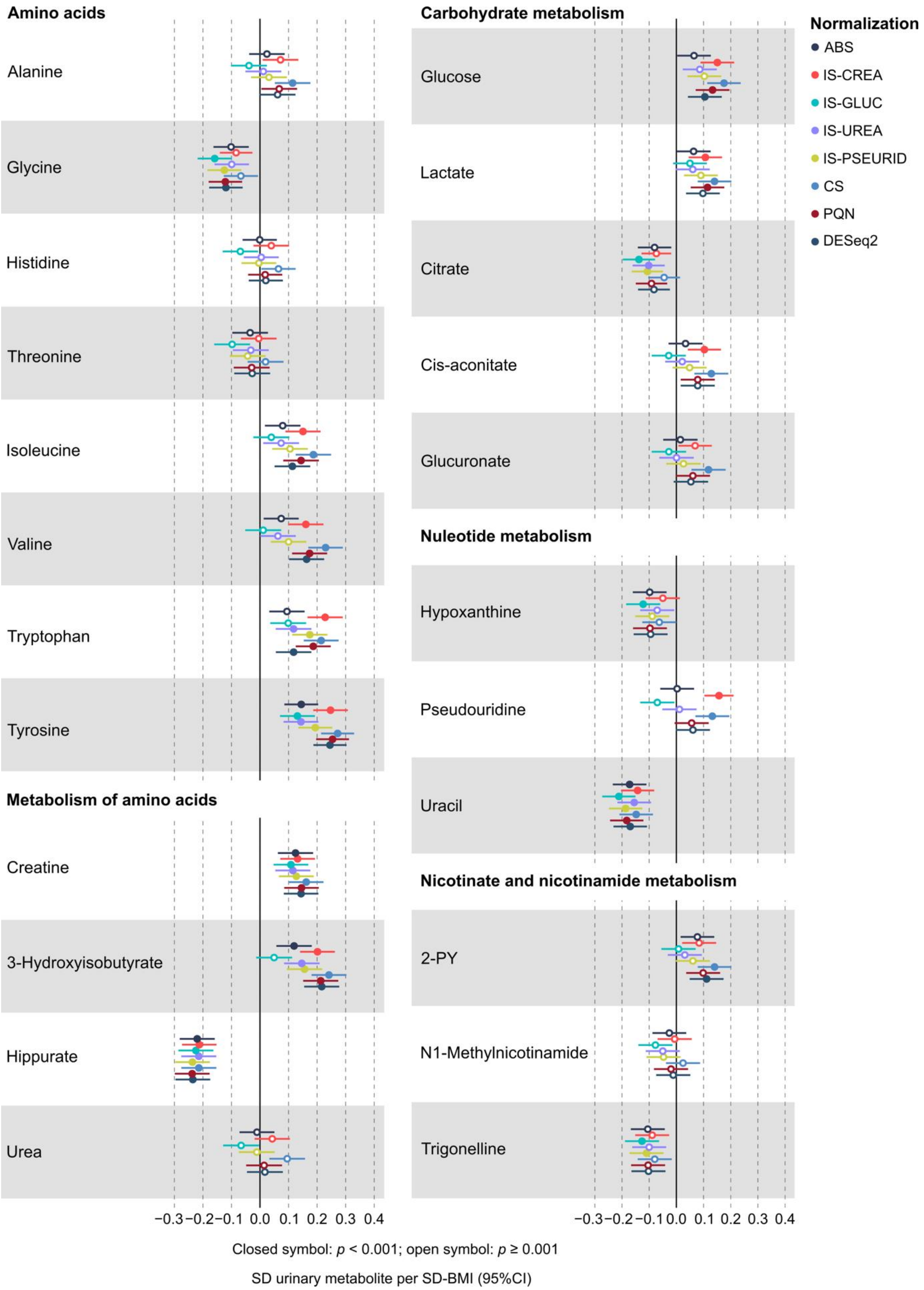

Continued on the next page

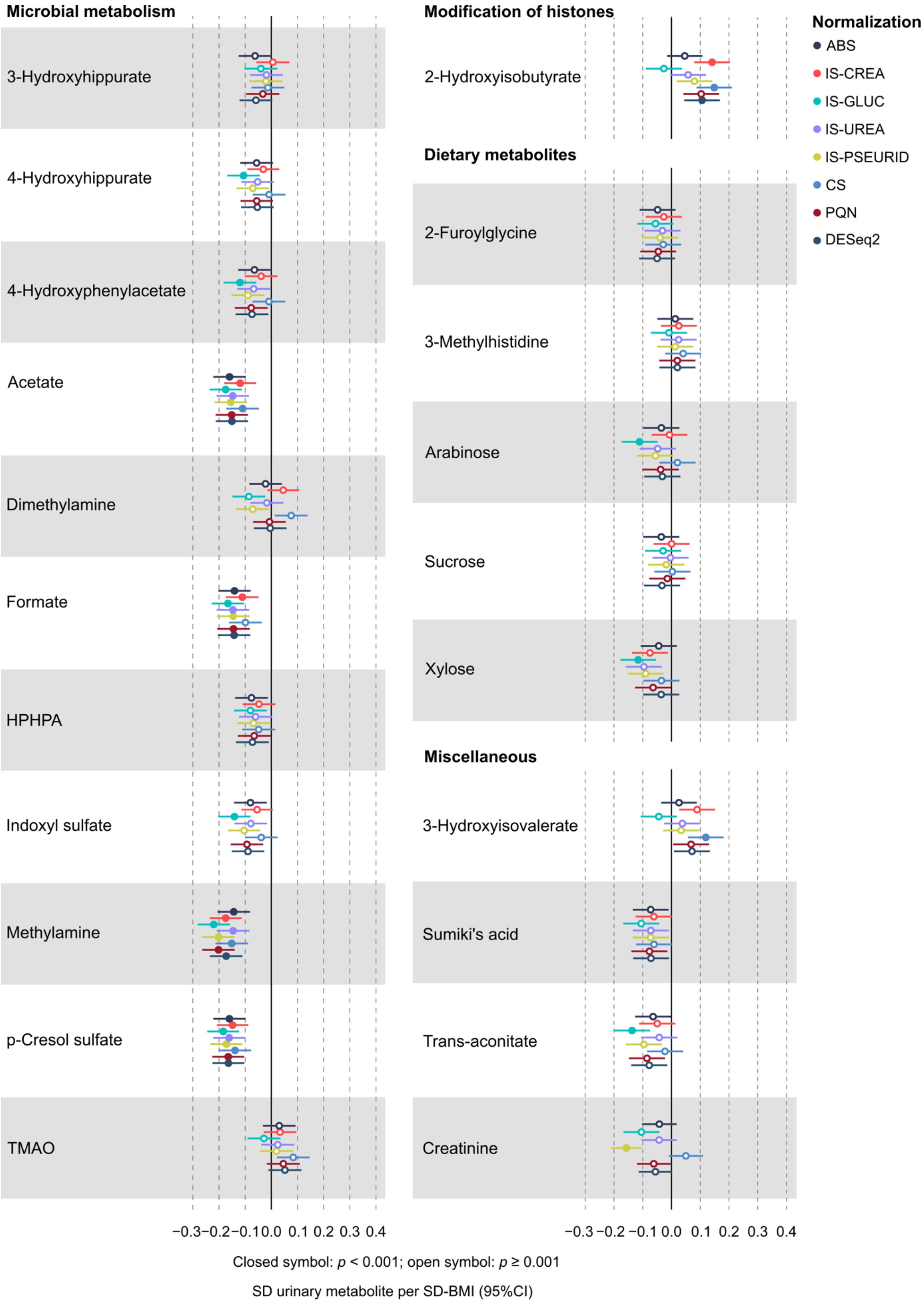

Figure S6. The associations of the urinary metabolite concentrations with BMI (adjusted for sex) for the various normalization schemes. Abbreviations: TMAO, trimethylamine N-oxide; HPHPA, 3-(3-hydroxyphenyl)-3-hydroxypropanoate; 2-PY, N1-Methyl-2-pyridone-5-carboxamide.

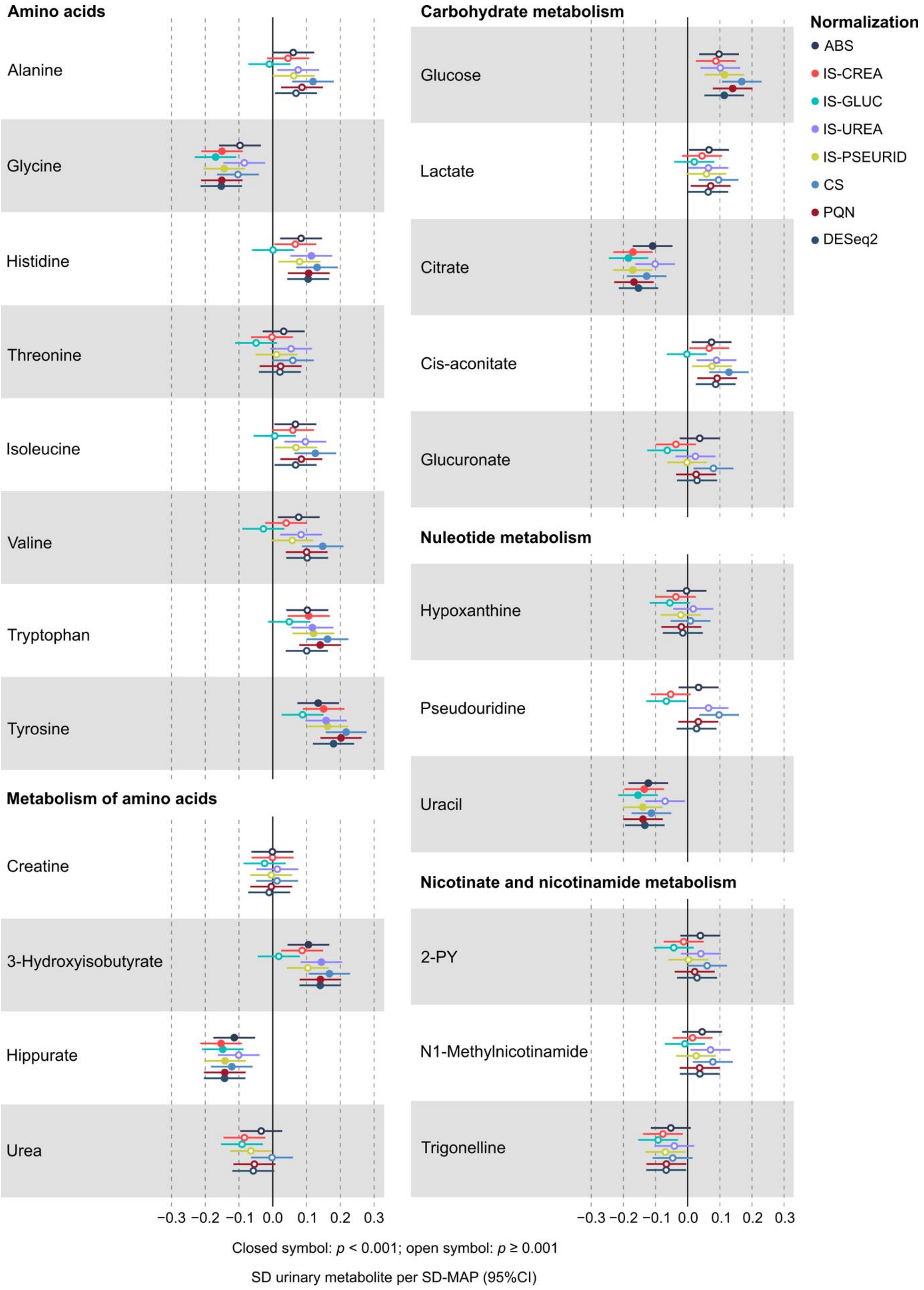

Continued on the next page

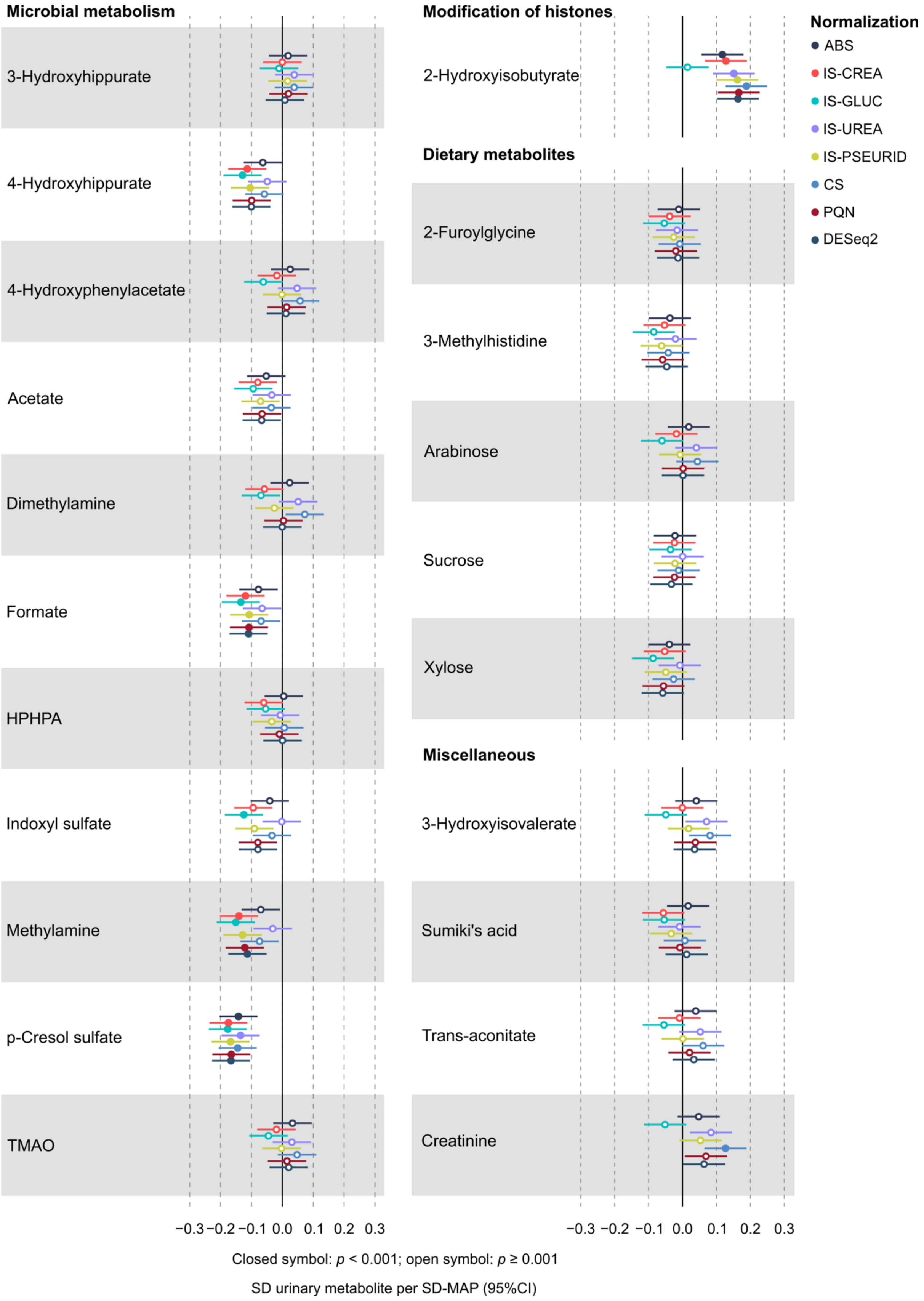

Figure S7. The associations of the urinary metabolite concentrations with MAP (without adjustments) for the various normalization schemes. Abbreviations: TMAO, trimethylamine N-oxide; HPHPA, 3-(3-hydroxyphenyl)-3-hydroxypropanoate; 2-PY, N1-Methyl-2-pyridone-5-carboxamide.

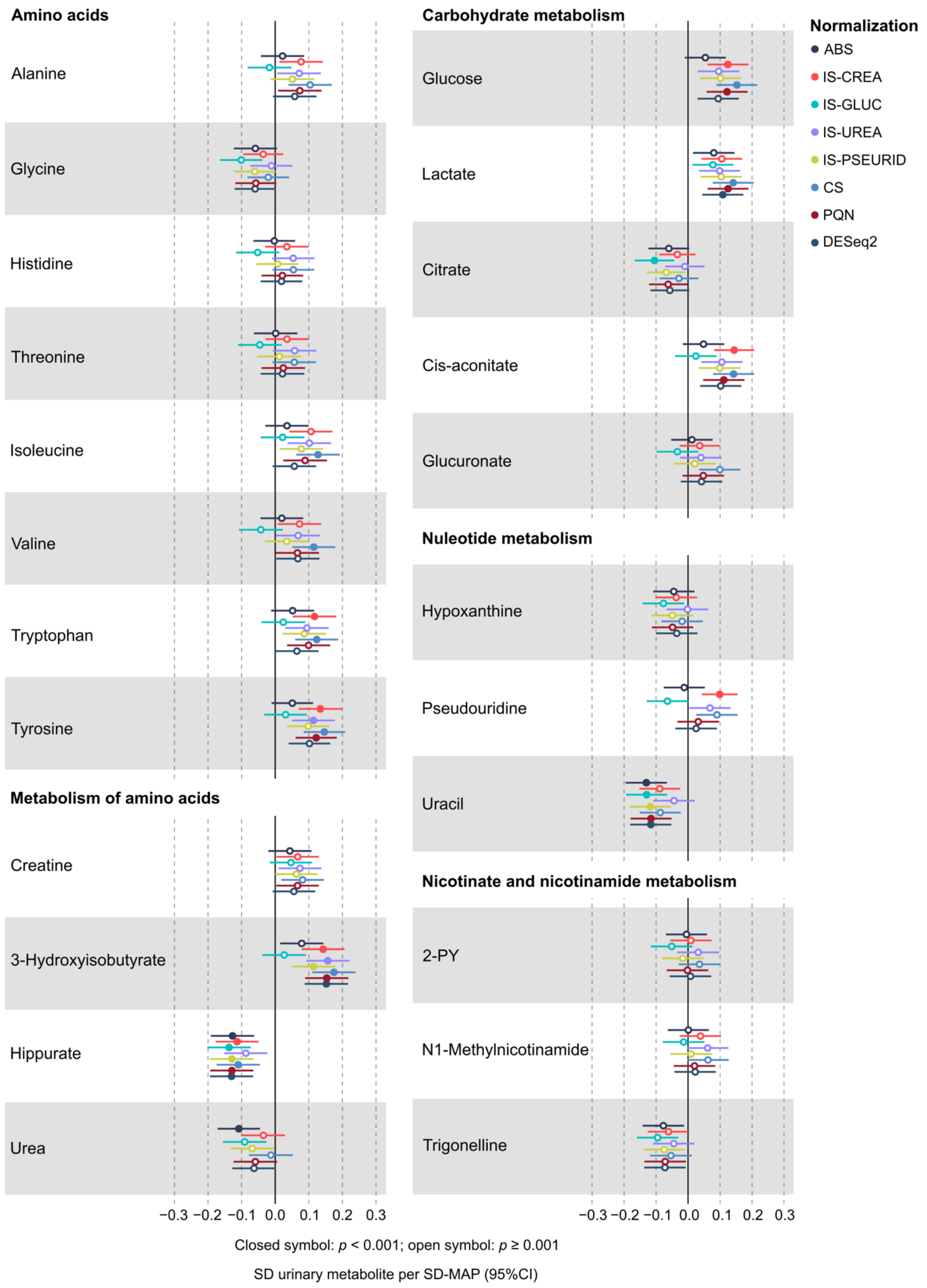

Continued on the next page

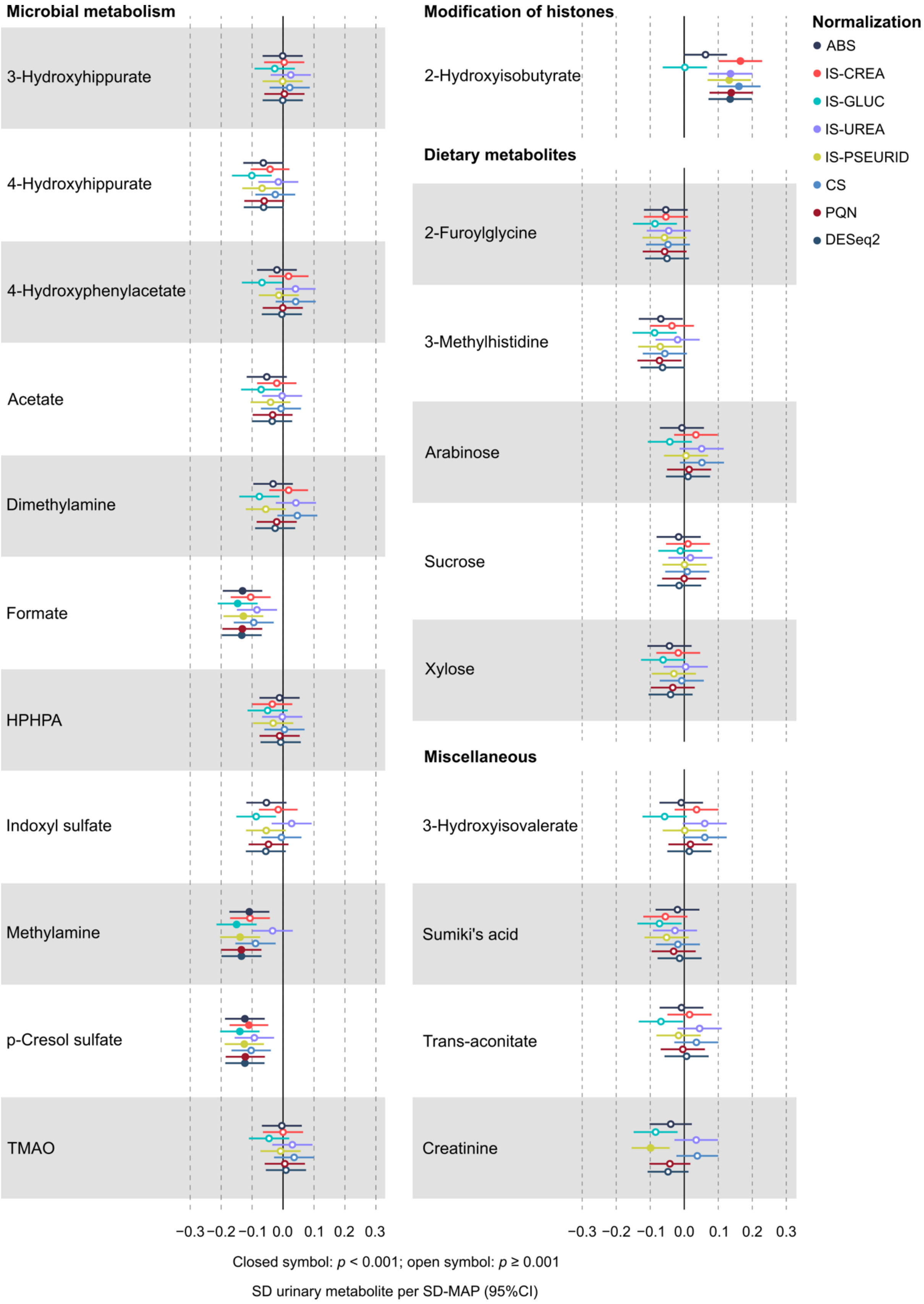

Figure S8. The associations of the urinary metabolite concentrations with MAP (adjusted for sex) for the various normalization schemes. Abbreviations: TMAO, trimethylamine N-oxide; HPHPA, 3-(3-hydroxyphenyl)-3-hydroxypropanoate; 2-PY, N1-Methyl-2-pyridone-5-carboxamide.

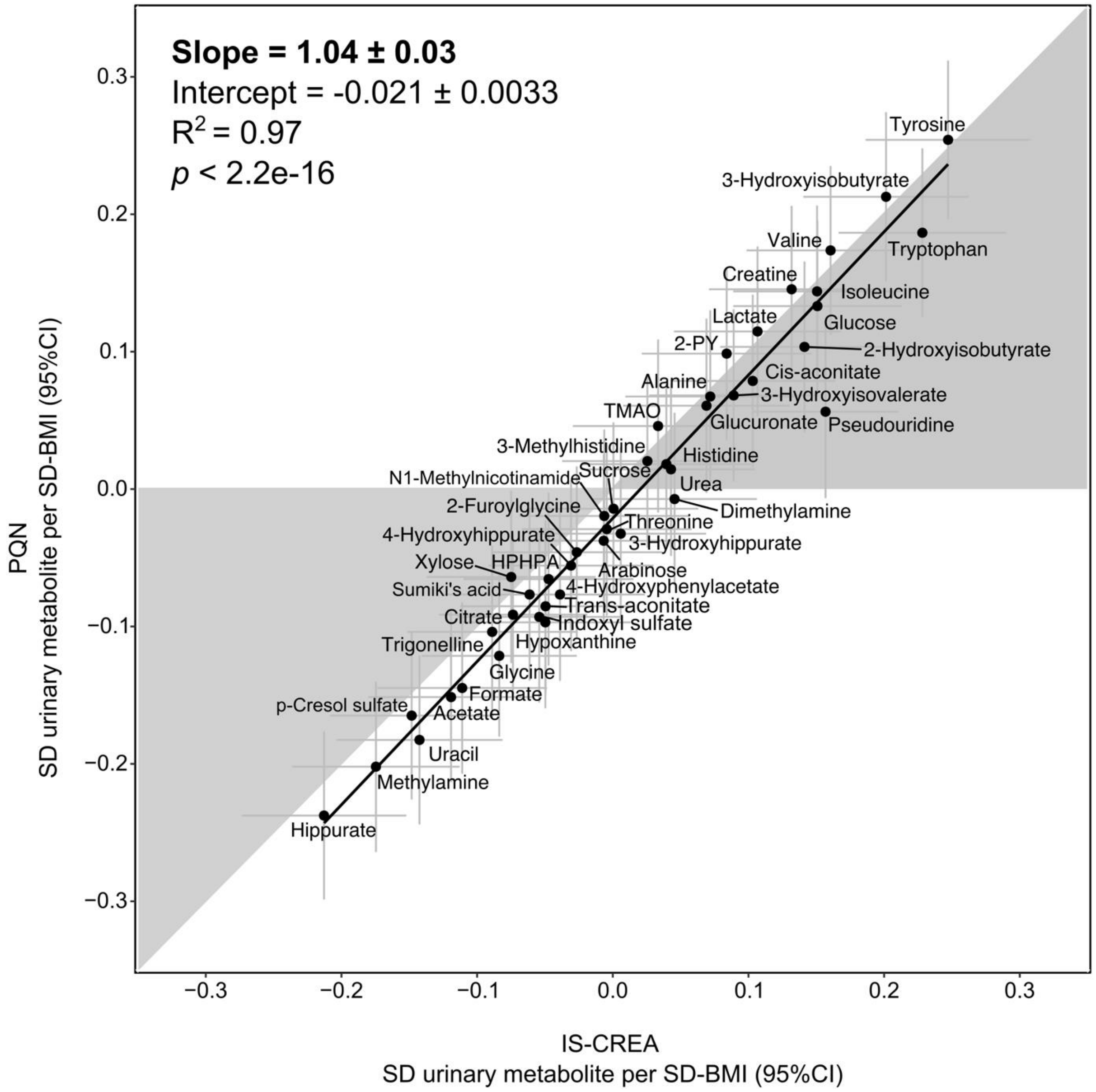

Figure S9. Comparison of the epidemiological associations of the urinary metabolite concentrations via IS-CREA and PQN normalization with BMI. Abbreviations: TMAO, trimethylamine N-oxide; HPHPA, 3-(3-hydroxyphenyl)-3-hydroxypropanoate; 2-PY, N1-Methyl-2-pyridone-5-carboxamide.
